# Intravitreal homocysteine-thiolactone injection leads to the degeneration of multiple retinal cells, including photoreceptors

**Published:** 2011-07-19

**Authors:** Han-Hsin Chang, David Pei-Cheng Lin, Ying-Shan Chen, Hsiang-Jui Liu, Wei Lin, Zih-Jay Tsao, Mei-Ching Teng, Bo-Yie Chen

**Affiliations:** 1 School of Nutrition, Chung Shan Medical University, Taichung, Taiwan, ROC; 2 Department of Ophthalmology, Chung Shan Medical University Hospital, Taichung, Taiwan, ROC; 3 School of Optometry, Chung Shan Medical University, Taichung, Taiwan, ROC; 4 School of Medical Laboratory and Biotechnology, Chung Shan Medical University, Taichung, Taiwan, ROC; 5 Department of Ophthalmology, Hsinchu Cathay General Hospital, Hsinchu, Taiwan, ROC; 6 Department of Optometry, Jen-Teh Junior College of Medicine, Nursing and Management, Miaoli, Taiwan, ROC; 7 Department of Optometry, Central Taiwan University of Science and Technology, Taichung, Taiwan, ROC; 8 Department of Ophthalmology, Kaohsiung Chang Gung Memorial Hospital and Chang Gung University College of Medicine, Kaohsiung, Taiwan, ROC

## Abstract

**Purpose:**

Hyperhomocysteinemia is known to cause degeneration of retinal ganglion cells, but its influence on photoreceptors remains largely unknown. In particular, the role of homocysteine-thiolactone (Hcy-T)—the physiologic metabolite of homocysteine that has been proven to be more cytotoxic than homocysteine itself—as a factor that causes retinopathy, has not been defined. This study aimed to investigate the toxic effects of excessive Hcy-T in a mouse model.

**Methods:**

A total of 60 six-week-old female ICR mice were used in this study. The mice were divided into 3 experimental groups and 2 control groups. The mice in the experimental groups were subjected to intravitreal injections of Hcy-T to reach final estimated intravitreal concentrations at 5, 25, and 200 μM, respectively. Mice without injection (blank) and with 0.9 NaCl injections (sham injection) were used as controls. The mice with 200 μM Hcy-T were sacrificed at days 7, 15, 45, and 90 after injection and the mice with 5 or 25 μM Hcy-T were sacrificed at day 90, with the controls sacrificed at day 15 or 90 for comparison. Semi-quantitative dot-blot analysis was performed for confirmation of retinal homocysteinylation. The mouse retinas were evaluated microscopically, with the thickness of total and specific retinal layers determined. Immunohistochemical analysis was performed and the labeled cells were quantified to determine the effects of excessive Hcy-T on specific retinal cells.

**Results:**

Dose-dependent retinal homocysteinylation after Hcy-T injection was confirmed. The homocysteinylation was localized in the outer and inner segments of photoreceptors and the ganglion cell layer (GCL). Retinal cell degenerations were found in the GCL, inner nuclear layer, and outer nuclear layer at day 90 after 200 µM Hcy-T injection. Significant thickness reduction was found in the total retina, outer nuclear layer, and the outer and inner segment layers. A trend of thickness reduction was also found in the GCL and inner nuclear layer, although this was not statistically significant. The rhodopsin^+^ photoreceptors and the calbindin^+^ horizontal cells were significantly reduced at day 15, and were nearly ablated at day 90 after 200 μM Hcy-T injection (p<0.001 for both day 15 and day 90), which was not seen in the sham injection controls. The Chx-10^+^ or the Islet-1^+^ bipolar cells and the Pax-6^+^ amacrine cells were severely misarranged at day 90, but no significant reduction was found for both cell types. The GFAP^+^ Müller cells were activated at day 15, but were not significantly increased at day 90 after the injection.

**Conclusions:**

Excessive retinal homocysteinylation by Hcy-T, a condition of hyperhomocysteinemia, could lead to degeneration of photoreceptors, which might lead to retinopathies associated with severe hyperhomocysteinemia or diabetes mellitus.

Received: April 1, 2011

Accepted: July 14, 2011

## Introduction

Homocysteine (Hcy) is a sulfur-containing amino acid derived from methionine metabolism as an intermediate metabolite. Clinically, the normal range of total plasma Hcy falls between 5 to 15 μmol/l. Patients with Hcy concentrations greater than 15 μmol/l are considered to have hyperhomocysteinemia (hHcy) and the condition is commonly associated with diabetes mellitus [1]. In recent years, hHcy has been implicated in systematic hypertension, stroke, and other cardiovascular diseases [2,3]. In the ocular system, many lines of evidence indicated hHcy as a risk factor in a variety of diseases, including retinal arteriosclerosis [4], glaucoma [5,6], exudative age-related macular degeneration [7], and macular and optic atrophy due to retinal vascular occlusion or non-arteritic ischemic optic neuropathy [8–10].

Notably, most hHcy-induced retinopathies are indirectly due to retinal arteriosclerosis and thrombosis. At the cellular level, previous studies have indicated involvement of hHcy in vascular endothelial damage and proliferation of vascular smooth muscle cells [11,12]. At the molecular level, hHcy was reported to be associated with elevated lipid peroxidation, endoplasmic reticulum stress, DNA methylation, and collagen accumulation [13,14]. Direct Hcy attack on retina has also been reported. Ganapathy et al. [15] used the cystathionine beta-synthase (cbs) mutant mouse to study the effects of endogenous homocysteine elevation on the retina. They found an approximate sevenfold elevation of Hcy in the cbs^−/−^ retina. As a result of Hcy elevation, distinct alterations were observed in the ganglion, inner plexiform, inner nuclear, and epithelial layers in retinas of cbs^−/−^ and 1-year-old cbs^+/−^ mice. High levels of Hcy were concomitantly located in these retinal layers, particularly the ganglion cell layer (GCL), suggesting direct attack by Hcy [15,16]. Another study by Lee et al. [17] showed that short-term hHcy-induced oxidative stress can activate retinal glial cells and increase VEGF expression in the retina.

The accumulation of Hcy in human cells depends on the activities of methionine synthase, cystathionine β-synthase, and methionyl-tRNA synthetase. Hcy can be metabolized by methionine synthase through its activity to convert Hcy into methionine. Hcy can also be converted into cysteine by cystathionine β-synthase. These two pathways deplete intracellular Hcy in a healthy manner. A third metabolic pathway for Hcy is its conversion into homocysteine -thiolactone (Hcy-T) by methionyl-tRNA synthetase, which occurs under normal physiologic reactions in all human cell types investigated [18]. Both Hcy and Hcy-T can attack proteins through either S-homocysteinylation or N-homocysteinylation [2]. S-homocysteinylation is undertaken by Hcy through attack on the Cys-SH group of proteins, while N-homocysteinylation involves acylation of Lys ε-amino group by the activated carboxyl group of Hcy-T [1,19]. Both types of homocysteinylation will cause protein damage and will lead to impairment of protein function [2]. As such, retinal proteins are liable to be attacked by homocysteinylation under the influence of hHcy. Besides, Hcy-T has been reported to possess stronger cytotoxicity and pro-inflammatory properties than Hcy [20]. Therefore, the third metabolic pathway for Hcy will potentially lead to more severe retinopathies. Yet, there has been no report regarding the direct role of Hcy-T in causing retinopathies.

Another interesting, important, and unanswered question is how hHcy can attack retina under in vivo conditions. There has so far been no report on hHcy-induced photoreceptor damage, although an earlier report indicated a non-detectable electroretinogram in a case of combined methylmalonic aciduria and homocystinuria [21]. Another report also showed some abnormalities on behavior tests assessing visual function in six cases of combined methylmalonic aciduria and homocystinuria [22]. Potentially, these clinical symptoms can be a result of damage to the photoreceptors and to the ganglion cells, under the influence of hHcy. Yet, there has been no data reported on this area.

In this study, we conducted intravitreal Hcy-T injections in a mouse model to investigate the direct role of Hcy-T in causing retinopathies. Following the injections, Hcy-T remained detected at day 90 in the retina in a dose-dependent manner, suggesting occurrence of homocysteinylation. The toxic level of intravitreal Hcy-T started at 25 μM in this mouse model. The ganglion cell layer (GCL), inner nuclear layer (INL), outer nuclear layer (ONL), and the outer segment and inner segment (OS/IS) of photoreceptors were severely affected. With intravitreal Hcy-T level at 200 μM, the ONL were all eradicated at day 90 after the injection. Total retinal thickness was reduced as compared to that of the blank or the sham injection controls. This reduction was also observed in the GCL, INL, ONL, and OS/IS layers, respectively. Immunohistochemical staining localized Hcy-T, mainly in the GCL and OS/IS layers, which persisted at day 90 after the injection. Photoreceptor and horizontal cells were the most affected as indicated by the disappearance of their specific markers. The bipolar, amacrine, and Müller glial cells were all disorganized. Our results indicate that excessive Hcy-T leads to more extensive retinal homocysteinylation than hHcy and most retinal cells, including photoreceptors, are damaged under the influence of excessive Hcy-T.

## Methods

### Animals

A total of 60 six-week-old female ICR mice were used in this study. The mice were purchased from National Laboratory Animal Center (Taipei, Taiwan, ROC). The mice weighed about 25 g on arrival and were fed ad libitum and kept under standard conditions with a 12 h:12 h light-dark cycle. The mice were acclimatized and habituated to the laboratory for at least one week before the experiments. All animal maintenance and experiment protocols were reviewed and approved by the Animal Care and Use Committee of Chung Shan Medical University and were performed in agreement with the Association for Research in Vision and Ophthalmology (ARVO) Resolution on the Use of Animals in Research.

### Study groups and intravitreal homocysteine-thiolactone injections

**Figure 1:**
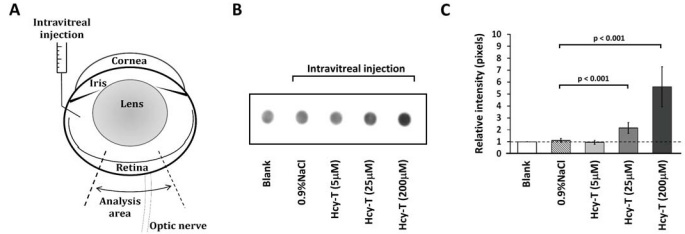
The preparation of intravitreal homocysteine-thiolactone (Hcy-T) injection is illustrated in **A** and retinal homocystinylation as confirmed by a semi-quantitative dot-blot analysis is shown in **B** and **C**. **A**: In each retinal section, the thickness measurement was made at two sites, located approximately 200 to 300 μm apart, on either side of the optic nerve. **B**: Dose-dependent retinal homocystinylation was observed on day 90 after injection. **C**: Quantification of relative intensity (pixels) on dot-blot membranes using an arbitrary unit of retinal homocystinylation as compared to that of the blank control. The sample size was 5 in all groups. The error bars indicate standard error of the means (SEMs).

Prior to intravitreal injection, the mice were anesthetized with intraperitoneal injections of sodium pentobarbital (45 mg/kg bodyweight). Intravitreal injection procedures and criteria were applied following the details published by Moore [23]. Briefly, both eyes were injected using a 5 μl Hamilton glass syringe (Hamilton Bonaduz AG, Bonaduz, Switzerland). The injections were made posterior to the iris and into the vitreous under a Nikon SMZ-645 dissection microscope (Nikon, Tokyo, Japan; Figure 1A). Caution was taken to avoid direct contact of the needle with the lens. The mice were injected intravitreally with either 0.9% NaCl (sham injection control) or Hcy-T (Sigma-Aldrich, St. Louis, MO) and some mice were left without injection to serve as blank controls (Table 1). The Hcy-T was prepared in 0.9% NaCl, pH 7.4. The final intravitreal concentration of Hcy-T was calculated based on an assumed vitreal volume at 25 μl per eye. Hcy-T was prepared in three stock solutions of 0.25, 1.25, and 10 mM. The injection of 0.5 μl into the vitreous gave a final estimated intravitreal concentration of 5, 25, and 200 μM, respectively. Since the purpose of this study was to assess the consequences of excessive levels of Hcy-T on the retinal cells, the intravitreal Hcy-T concentrations tested in this study were set above a physiologic range or were equivalent to those of the retinopathogenetic conditions [24,25].

**Table 1:** Summary of experiments

**Numbers**	**Days after injection when sacrificed**	**Number of mice**	**Final intravitreal concentrations**
1	90	8	Non-injection control (Blank control)
2	15	5	0.9% NaCl
3	90	8	0.9% NaCl
4	90	8	Homocysteine thiolactone (5 μM)
5	90	8	Homocysteine thiolactone (25 μM)
6	7	5	Homocysteine thiolactone (200 μM)
7	15	5	Homocysteine thiolactone (200 μM)
8	45	5	Homocysteine thiolactone (200 μM)
9	90	8	Homocysteine thiolactone (200 μM)

### Semi-quantitative dot-blot analysis for retinal homocysteinylation

Dot-blot analysis was used to test the levels of retinal homocysteinylation after intravitreal Hcy-T injections. After the mice were euthanatized, their eyes were extracted, followed by removal of the cornea and the lens tissues and separation of the retinas from the eye cups. The retinas were homogenized in radioimmunoprecipitation (RIPA) buffer (Upstate Biotechnology, Lake Placid, NY) containing a protease inhibitor cocktail and were then centrifuged at 10,000× g for 15 min at 4 °C. The supernatants were collected and assayed for their protein levels with a Bio-Rad Protein Assay kit (Life Science Research, Hercules, CA) using BSA as standard. Equal aliquots (30 μg) of the retinal proteins were applied to a 96-well dot blot apparatus (Life Science Research) and were then transferred to a nitrocellulose membrane by vacuum filtration. The preparation was immersed in 10% non-fat milk for 30 min at room temperature and was then incubated with the rabbit anti-homocysteine antibody (1/1500, cat. ab15154; Abcam, Cambridge, MA) overnight at 4 °C, followed by incubation with the peroxidase-linked antirabbit IgG antibody (1/1000; Jackson ImmunoResearch Laboratories, Inc., West Grove, PA) for 1 h at room temperature. After thorough washing, chemiluminescence was developed (Immobilon Western chemiluminescent horseradish peroxidase substrate; Millipore, Temecula, CA) and detected with a digital imaging system (IS4000R; Kodak, New Haven, CT). Intensities of dots stained with anti-homocysteine were determined by calibration of the total amounts of pixels using the ImageJ image processing program (National Institute of Health, Bethesda, MD). The retinal homocysteinylation levels in the injection groups were represented as the relative amounts of pixels compared with those of the retinas without injection (blank controls). Six independent samples from each injection group were determined. To ensure independent observation, only one eye from each mouse was randomly selected for assessment of retinal homocysteinylation.

### Microscopic evaluation and quantification of thickness for total and specific retinal layers

For microscopic evaluations, the mice were sacrificed by cervical dislocation. One eye from each mouse was randomly selected and extracted. The extracted eyes were fixed in 4% formalin for at least 24 h, washed with 0.9% NaCl, and processed through ethanol and xylene solutions. The preparations were then embedded in paraffin, cut at 5-μm thickness, and mounted on glass slides following conventional procedures [26–28]. Hematoxylin-Eosin stain was performed for histopathological examinations [28,29]. The retinal tissue sections were scanned for evidence of gross defects and were then used to measure the thickness of the total retina, GCL, INL, ONL, and the OS/IS of the photoreceptors. In each retinal section, the measurement of thickness was made on two sites, approximately 200 to 300 μm apart, on either side of the optic nerve. The results of these measurements were then averaged for that particular section. For each retina analyzed, three separate eye sections were measured by two observers without prior knowledge of the study groups. All measurements were performed with a Nikon E100 microscope (Nikon,) equipped with a digital camera and the ImageJ image processing program (National Institute of Health).

### Immunohistochemical analysis and quantification of labeled cells

For immunohistochemical analysis, the retinal tissue sections were boiled in a citrate buffer (pH 6.0) for 20 min for antigen retrieval and were then incubated, respectively, with one of the following antibodies: rabbit anti-homocysteine (1/50, cat. ab15154; Abcam); mouse anti-rhodopsin (1/400, cat. Ab81702; Abcam); mouse anti-calbindin (1/50, cat. sc-74462; Santa Cruz Biotechnology, Santa Cruz, CA); mouse anti-Pax-6 antibody (1/50, cat. sc-32766; Santa Cruz Biotechnology); rabbit anti-Islet-1 (1/150, cat. Ab20670; Abcam); sheep anti-Chx-10 (1/150, cat. ab16141; Abcam); or rabbit anti-GFAP (1/250, cat. 2301–1; Epitomics, Burlingame, CA). The preparations were then incubated with a horseradish peroxidase-conjugated secondary antibody (1/200), either antimouse, antirabbit, or antisheep IgG (Jackson ImmunoResearch Laboratories, Inc.). After incubation, the preparations were washed thoroughly, incubated in diaminobenzidine tetrahydrochloride solution for color detection, and were counterstained with hematoxylin. Positively labeled cells were counted on two sites, approximately 200 to 300 μm, apart on either side of the optic nerve. The areas for counting were fixed at 100 μm in width for each site. The Rhodopsin positive cells were counted in the ONL. The calbindin positive cells were counted in the OPL. The Islet-1 or Chx-10 or Pax-6 positive cells were counted in the INL. The glial fibrillary acidic protein (GFAP) positive cells were counted in the whole thickness of the retina. The results of counting were then averaged for that particular section. For each retina analyzed, three separate sections were measured by two observers without prior knowledge of the study groups. All quantifications of labeled cells were performed with a Nikon E100 microscope (Nikon) equipped with a digital camera and the ImageJ image processing program (National Institute of Health,).

### Statistical analysis

All data were obtained from triple repeats and are presented as the means±standard error of the means (SEMs) and were compared among groups. The retinal thickness and the means of labeled retinal cells were compared and analyzed by the Mann–Whitney test. All statistical analyses were performed using the Prism program (GraphPad Software, San Diego, CA).

## Results

### Retinal homocysteinylation after intravitreal homocysteine thiolactone injection

We first tried to determine whether intravitreal Hcy-T injection can lead to homocysteinylation in the retina. Dot-blot analysis was performed using the supernatant of the whole retina at day 90 after the injection. Retinal homocysteinylation was found in a dose-dependent manner, with the highest extent of homocysteinylation found in the retinas injected with Hcy-T to reach a final concentration of 200 μM (Figure 1B). Quantitative analysis on the relative density of dot blots showed that Hcy-T injections for final 200 μM resulted in approximately a fivefold increase in retinal homocysteinylation compared to the sham injection controls (p<0.001; Figure 1C). The results indicated that intravitreal Hcy-T injections could lead to retinal homocysteinylation and the doses of Hcy in the vitreous fluid will determine the extent of homocysteinylation.

### Retinal degeneration and reduced thickness of total retina and specific retinal layers after retinal homocysteinylation

**Figure 2:**
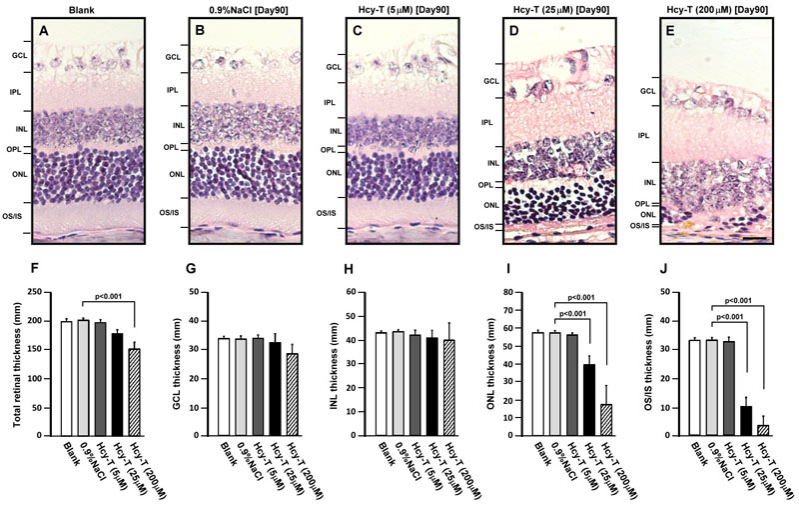
The effects of an intravitreal homocysteine-thiolactone (Hcy-T) injection on mouse retinas at day 90 after preparation. All retinas were prepared with hematoxylin-eosin staining for histological assessment and for measurement of the thickness of specific layers. **A**: Blank control without any injection. **B**: 0.9% NaCl sham injection control. **C**: Final intravitreal Hcy-T at 5 μM. **D**: Final intravitreal Hcy-T at 25 μM. **E**: Final intravitreal Hcy-T at 200 μM. **F**: Total retinal thickness. **G**: Ganglion cell layer (GCL) thickness. **H**: Inner nuclear layer (INL) thickness. **I**: Outer nuclear layer (ONL) thickness. **J**: Outer and inner segments of photoreceptors (OS/IS) thickness. The sample size for total retinal thickness was 5 in all groups. The error bars indicate standard error of the means (SEMs). Scale bar: 25 μm.

With retinal homocysteinylation confirmed after Hcy-T injections for final concentrations of 5, 25, and 200 μM, we then examined the effects after this manipulation. There was no evidence of the degeneration of specific retinal layers following Hcy-T injection for 5 μM. Hcy-T injection for 25 μM, however, started to result in retinal cell degeneration, particularly in the GCL, INL, and ONL, where most of the retinal cell nuclei are located (Figure 2D). Furthermore, the inner plexiform layer (IPL) generally became loosely deployed, suggesting occurrence of edema. Notably, all of the cells in the GCL, INL, and ONL exhibited nucleus condensation and lost their orderly organization as a layer. Quantitative analysis showed a trend of thickness reduction in the total retina, with significant difference found in the group injected with 200 μM of Hcy-T, as compared to that of the sham injection control group (Figure 2F). With Hcy-T injection, the GCL and INL layers were also reduced in thickness, although the results were not statistically significant (Figure 2G,H). In contrast, the thickness of the ONL and OS/IS layers were already significantly reduced after Hcy-T injection for 25 μM, and Hcy-T injection for 200 μM lead to additional reduction (both p<0.001; Figure 2I,L). These results indicated that Hcy-T injections caused reduction of total retinal thickness, which was likely due to the effects on multiple retinal layers.

### Localization of retinal homocysteinylation after Hcy-T injection

**Figure 3:**
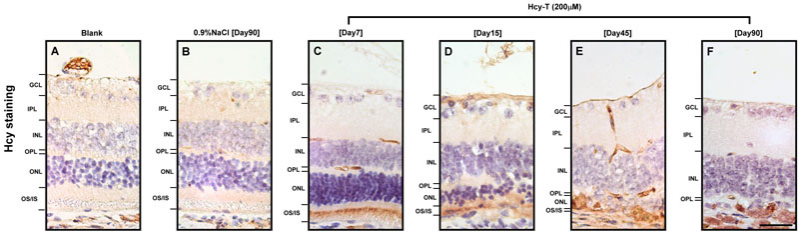
The immunohistochemical localization of retinal homocystinylation sites at days 7, 15, 45, 90 after a homocysteine-thiolactone (Hcy-T) injection to reach a final intravitreal Hcy-T concentration at 200 μM. **A**: Blank control without any injection. **B**: 0.9% NaCl sham injection control at day 90. **C**: Day 7 after injection. **D**: Day 15 after injection. **E**: Day 45 after injection. **F**: Day 90 after injection. Scale bar: 40 μm.

To further understand the effects of excessive Hcy-T on the retina, we compared the localization of retinal homocysteinylation by immunohistochemistry at days 7, 15, 45, and 90 after injections for a final intravitreal Hcy-T concentration at 200 μM. Generally, the sites of retinal homocysteinylation were most evident in the OS/IS and the GCL layers (Figure 3C-F), as compared to the absence of evident localization in the blank and the sham injection controls (Figure 3A,B). Evidence of accumulation of the wreckage of degenerated photoreceptors was seen on day 45 and day 90 after Hcy-T injection (Figure 3E-F).

### The effect of intravitreal Hcy-T injections on specific retinal cell types

**Figure 4:**
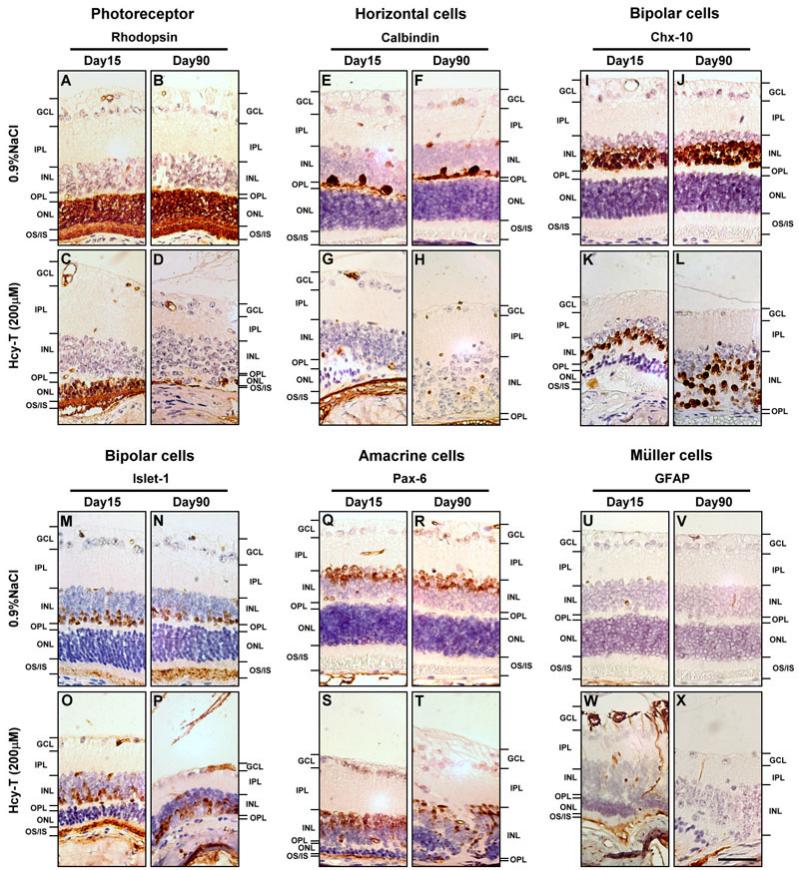
The cytotoxic effects of homocysteine-thiolactone (Hcy-T) on specific retinal cells as reflected by alterations of **A**-**D**: rhodopsin positive photoreceptors, **E**-**H**: calbindin positive horizontal cells, **I**-**L**: Chx- 10 positive bipolar cells, **M**-**P**: Islet-1 positive bipolar cells, **Q**-**T**: Pax-6 positive amacrine cells, and **U**-**X**: glial fibrillary acidic protein (GFAP) positive Müller cells at day 15 and day 90 following the intravitreal injections. Abbreviations: GCLrepresents ganglion cell layer; INL represents inner nuclear layer; IPL represents inner plexiform layer; ONL represents outer nuclear layer; OPL represents outer plexiform layer; OS/IS represents outer and inner segments of photoreceptors. Scale bar: 45 μm.

Since the thickness reduction was observed in multiple layers of the retina, it is important to identify the specific retinal cell types affected after intravitreal Hcy-T injection for 200 μM. We therefore conducted immunohistochemistry using specific markers for photoreceptors (rhodopsin), horizontal (calbindin), bipolar (Chx-10 or Islet-1), amacrine (Pax-6), and Müller cells (GFAP) at day 15 and day 90 following the injections. At day 15, the rhodopsin^+^ photoreceptor cells were significantly reduced (Figure 4C) as compared to those of the sham injection control (Figure 4A). This reduction of photoreceptors was aggravated to the extent that almost all photoreceptors were ablated at day 90 after the injection (Figure 4D), which was not seen in the sham injection controls (Figure 4B). The calbindin^+^ horizontal cells were also affected at day 15 after the injection (Figure 4G) and were also totally ablated in most retinas at day 90 after the injection (Figure 4H), in contrast to the normal presence and deployment of horizontal cells in the sham injection controls at day 15 (Figure 4E) and day 90 (Figure 4F), respectively. We used two markers, Chx-10 and Islet-1, for the identification of bipolar cells (Figure 4I,P). At day 15 after the Hcy-T injection, misarrangement of bipolar cells was readily found (Figure 4K,O), which was not found in the sham injection controls (Figure 4I,M). The same misarrangement of bipolar cells was also observed at day 90 after the Hcy-T injection (Figure 4L,P), in contrast to the normal deployment of bipolar cells seen in the sham injection controls at the same stage (Figure 4J,N). The Pax-6^+^ amacrine cells appeared normal at day 15 after the injection (Figure 4S), but they were generally misarranged at day 90 (Figure 4T), as compared to the sham injection controls at day 15 (Figure 4Q) and day 90 (Figure 4R). The GFAP^+^ Müller cells were activated at day 15 after the injection (Figure 4W), which was not found in the sham injection controls (Figure 4U). However, the activated Müller cells were generally not seen at day 90 after the injection (Figure 4X), as compared to the results in the sham injection control group (Figure 4V).

**Figure 5:**
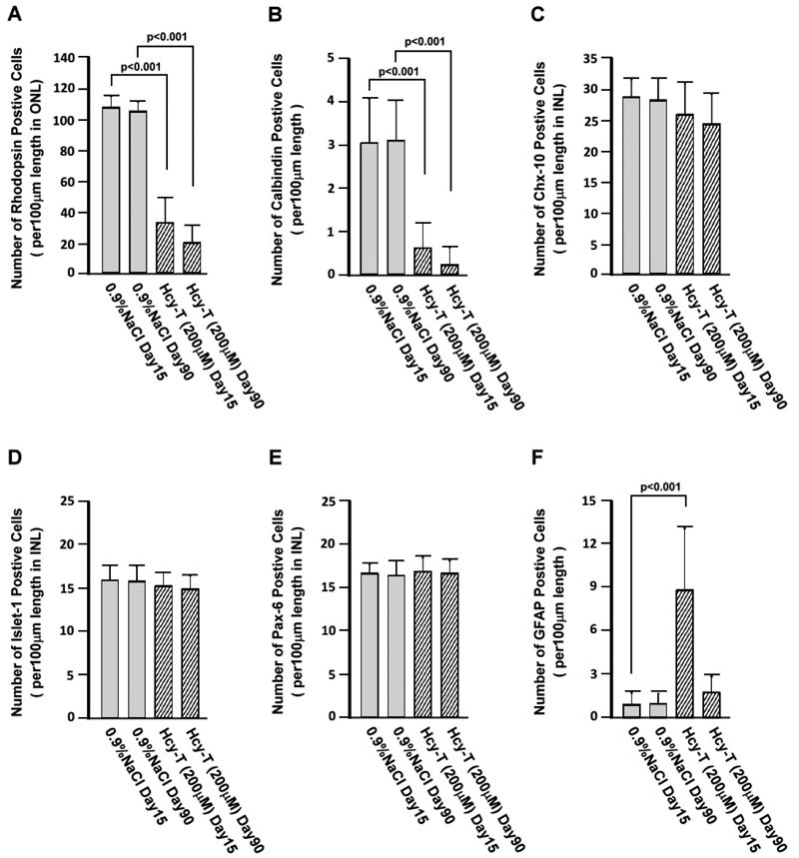
Quantification of specific retinal cells per 100 μm length according to their specific markers. The markers were used to detect at day 90 following the intravitreal injections. **A**: Rhodopsin positive cells per 100 μm length in outer nuclear layer (ONL). **B**: Calbindin positive cells per 100 μm length in the retina. **C**: Chx-10 positive cells per 100 μm length in inner nuclear layer (INL). **D**: Islet-1 positive cells per 100 μm length in INL. **E**: Pax-6 positive cells per 100 μm length in INL. **F**: Glial fibrillary acidic protein (GFAP) positive cells per 100 μm length in the retina. The sample size for all markers was 5 in all groups. The error bars indicate standard error of the means (SEMs).

To further confirm our observations on the histological level, we quantified specific retinal cell types according to the expression of their markers. At day 15 after intravitreal Hcy-T injections, the cell populations of rhodopsin^+^ photoreceptor cells and calbindin^+^ horizontal cells were both significantly reduced (Figure 5A,B; p<0.001 for both), as compared to those of the sham injection controls. Significant reduction of the rhodopsin^+^ photoreceptor cells and the calbindin^+^ horizontal cells was also seen at day 90 (p<0.001 for both). Ruction of cell populations was also observed in the Chx-10^+^ (Figure 5C) or the Islet-1^+^ (Figure 5D) bipolar cells, but not in the Pax-6^+^ amacrine cells (Figure 5E). Interestingly, the GFAP^+^ Müller cells were significantly activated at day 15 after intravitreal Hcy-T injections, but the activation was not seen at day 90 as there was no significant increase in the numbers of Müller cells detected (Figure 5F). The results of the quantification of specific cell types in the retinas after intravitreal Hcy-T injection confirmed our observations on the tissue level.

## Discussion

Hyperhomocysteinemia (hHcy) has been implicated in human cardiovascular and neurodegenerative diseases, particularly under diabetic conditions [2,3,16]. In recent years, many lines of evidence have indicated high levels of Hcy in the plasma or vitreous fluid [24,25,30] or deficiency of methionine synthase [22] as risk factors of clinical retinopathies and disturbed visual functions [24,31–33]. Hcy, like the neurotransmitter amino acid glutamate, is excitotoxic when its plasma concentration is elevated [13,14]. Hcy-T, the metabolically active form of Hcy, is known to be more cytotoxic than Hcy itself [1,2,18,19] and increased Hcy-T levels have been associated with severe diabetic retinopathy [30]. In previous literature, although the neurotoxic effects of Hcy-T and Hcy on retinal ganglion cells have been reported [15,16,23,34,35], little is known about their toxic effect on photoreceptors and other retinal cells. Clinically, however, a methionine synthase deficiency child with severe hyperhomocysteinemia was reported to show photopic ERG responses related to impaired photoreceptor function [36]. Earlier reports had also associated the combined conditions of methylmalonic aciduria and homocystinuria with abnormal electroretinograms and visual function [21,22]. Thus, in this study, we established a mouse hyperhomocysteinemia model to determine the effects of Hcy-T. The retinas administered with an intravitreal Hcy-T injection were analyzed and compared with their age-matched blank controls and sham injection controls.

Our present study leads to several important findings in support of photoreceptor degeneration induced by excessive Hcy-T. First, the thickness of the ONL and OS/IS of photoreceptors was reduced in the retinas following Hcy-T injections for final intravitreal concentrations at 25 μM and 200 μM, as compared to the age-matched 0.9%NaCl sham injection controls or the blank controls, suggesting photoreceptor cell loss in those layers. Second, the loss of photoreceptors was further supported by specific localization of retinal homocysteinylation on the photoreceptor OS/IS junction at day 7 after Hcy-T injection for 200 μM (Figure 3C), which likely started to damage the photoreceptors, resulting in the accumulation of the wreckage of degenerated photoreceptors as seen at day 45 and day 90 (Figure 3E-F). Third, another line of evidence also showed a significant loss of rhodopsin^+^ photoreceptors in the ONL after Hcy-T injection for 200 μM, as compared to the age-matched 0.9% NaCl sham injection controls (Figure 4A-D and Figure 5A).

As photoreceptors were found to be degenerated after Hcy-T injection, several important implications can be derived from this study. First, the integrity of the photoreceptor OS/IS layer in the retina is highly critical as vision stimuli are initially triggered when the photosensitive proteins in the outer segment absorb photons and generate a signal that will eventually be transmitted to the visual cortex. Notably, the junction region of OS/IS serves as the site for generation of new outer segment discs, where newly synthesized rhodopsins are incorporated. Clinically, the integrity of the photoreceptor OS/IS layer is known to correlate with visual function and visual acuity in patients with retinitis pigmentosa [37,38]. In this study, we observed reduced thickness of the OS/IS layers (Figure 2D,E,J) and loss of rhodopsin protein incorporation into the OS/IS layers (Figure 4C,D) at day 90 after Hcy-T injection. These effects are likely to be directly caused by homocysteinylation, since homocysteinylation was localized in the OS/IS layers (Figure 3C,D,F). When the OS/IS structures are targeted directly by homocysteinylation, the physiologic renewal process for installment of photosensitive proteins will be disrupted. Thus, the status of retinal homocysteinylation by Hcy-T might be regarded as an important risk factor for poor visual acuity or vision loss in clinical retinopathies associated with hyperhomocysteinemia. Second, the horizontal cells are second-order neurons of the vertebrate retina, being responsible for the lateral integration of signal transmission between photoreceptor and bipolar cells. Retinal cone photoreceptors have been reported to drive the horizontal cells to express N-methyl-D-aspartate (NMDA) receptors [39,40]. It has also been reported that overstimulation of NMDA receptors by excessive glutamate or homocysteine on the retinal ganglion cells in vitro leads the cells into a tract of rapid death [23,34]. Thus, the loss of horizontal cells after Hcy-T injection in vivo (Figure 4E-H and Figure 5B) might mediate the same pathway to cell death. This hypothesis, however, remains to be confirmed. Third, Hcy-T might induce endoplasmic reticulum (ER) stress and cause damage to photoreceptors, resulting in a reduction in ONL thickness (Figure 2D-E,I). This induced ER stress and cell death has been well documented in the vascular endothelial cells [41,42] and lens epithelial cells [43] under the influence of excessive Hcy-T or Hcy. Previous reports have implicated ER stress as a critical factor in retinal degeneration [44,45], but without referring to hyperhomocysteinemia. It is likely that the same mode of action occurs in the retina after Hcy-T injection.

Apart from photoreceptor degeneration, significant loss of horizontal cells (calbindin^+^ cells) in the OPL was also found after Hcy-T injection for 200 μM as compared to the age-matched 0.9% NaCl sham injection controls (Figure 4E-H and Figure 5B). Ruction of cell populations was also observed in the Chx-10^+^ (Figure 5C) or the Islet-1^+^ (Figure 5D) bipolar cells. The disruption process of the horizontal cells (calbindin^+^ cells) and bipolar cells after Hcy-T injection seemed to differ from that of the OS/IS photoreceptor layers. Hcy-T might directly target the OS/IS layers since homocysteinylation was localized there. In contrast, the disruption of the horizontal cells and bipolar cells appeared to be due to indirect effects, as no apparent homocysteinylation was localized within these cells. Functionally, bipolar and horizontal cells act as interneurons to transfer information from photoreceptors to ganglion cells [46]. Reduction or disruption of these interneurons will lead to lower sensitivity to contrast and differences in intensity, resulting in lower visual acuity [46–48]. Therefore, it is likely that hyperhomocysteinemia, commonly found in patients with diabetes mellitus [1,24,25], is a factor causing the reduced visual acuity found in diabetic retinopathy.

Interestingly, the GFAP^+^ Müller cells were significantly activated at day 15 after intravitreal Hcy-T injections, but the activation was not seen at day 90, as no significant increase in GFAP^+^ Müller cells was detected (Figure 5F). Müller cells contain multiple functions vital to the health of other retinal neuron cells [49,50]. They may be involved in both the phagocytosis of neuronal debris and the maintenance of other retinal cells. The early increase in GFAP^+^ Müller cells at day 15 may represent a functional response to deplete the damaged retinal cells after Hcy-T injection. This early effort obviously failed as the GFAP^+^ Müller cell population was reduced to nearly the same level as that in the sham-injection control at day 90. The result from this mouse model indicates the risk of chronic hyperhomocysteinemia in a period of just 90 days.

We conclude that excessive Hcy-T could cause degeneration of photoreceptors in the mouse retina. Our results support the necessity of evaluating photoreceptors and the OS/IS junction in patients with severe hyperhomocysteinemia or diabetes mellitus.
